# COM3/369: Knowledge-based Information Systems: A new approach for the representation and retrieval of medical information

**DOI:** 10.2196/jmir.1.suppl1.e16

**Published:** 1999-09-19

**Authors:** G Mann, C Birkmann, T Schmidt, V Schaeffler

**Affiliations:** ^1^GSF - medis Insitute, NeuherbergGermany

**Keywords:** Knowledge-based Information Systems, Knowledge-based Systems, Information Retrieval

## Abstract

**Introduction:**

Present solutions for the representation and retrieval of medical information from online sources are not very satisfying. Either the retrieval process lacks of precision and completeness the representation does not support the update and maintenance of the represented information. Most efforts are currently put into improving the combination of search engines and HTML based documents. However, due to the current shortcomings of methods for natural language understanding there are clear limitations to this approach. Furthermore, this approach does not solve the maintenance problem. At least medical information exceeding a certain complexity seems to afford approaches that rely on structured knowledge representation and corresponding retrieval mechanisms.

**Methods:**

Knowledge-based information systems are based on the following fundamental ideas. The representation of information is based on ontologies that define the structure of the domain's concepts and their relations. Views on domain models are defined and represented as retrieval schemata. Retrieval schemata can be interpreted as canonical query types focussing on specific aspects of the provided information (e.g. diagnosis or therapy centred views). Based on these retrieval schemata it can be decided which parts of the information in the domain model must be represented explicitly and formalised to support the retrieval process. As representation language propositional logic is used. All other information can be represented in a structured but informal way using text, images etc. Layout schemata are used to assign layout information to retrieved domain concepts. Depending on the target environment HTML or XML can be used.

**Results:**

Based on this approach two knowledge-based information systems have been developed. The 'Ophthalmologic Knowledge-based Information System for Diabetic Retinopathy' (OKIS-DR) provides information on diagnoses, findings, examinations, guidelines, and reference images related to diabetic retinopathy. OKIS-DR uses combinations of findings to specify the information that must be retrieved. The second system focuses on nutrition related allergies and intolerances. Information on allergies and intolerances of a patient are used to retrieve general information on the specified combination of allergies and intolerances. As a special feature the system generates tables showing food types and products that are tolerated or not tolerated by patients. Evaluation by external experts and user groups showed that the described approach of knowledge-based information systems increases the precision and completeness of knowledge retrieval. Due to the structured and non-redundant representation of information the maintenance and update of the information can be simplified. Both systems are available as WWW based online knowledge bases and CD-ROMs (cf. http://mta.gsf.de topic: products).

